# Translation and cross-cultural adaptation into Brazilian Portuguese of the Vanderbilt Head and Neck Symptom Survey version 2.0 (VHNSS 2.0) for the assessment of oral symptoms in head and neck cancer patients submitted to radiotherapy^☆^

**DOI:** 10.1016/j.bjorl.2015.08.014

**Published:** 2015-09-08

**Authors:** Eliane Marçon Barroso, André Lopes Carvalho, Carlos Eduardo Paiva, João Soares Nunes, Bianca Sakamoto Ribeiro Paiva

**Affiliations:** a Postgraduate Program in Oncology, Hospital de Câncer de Barretos, Barretos, SP, Brazil; b Head and Neck Department, Hospital de Câncer de Barretos, Barretos, SP, Brazil; c Grupo de Pesquisa em Qualidade de Vida Relacionada à Saúde (GPQual) – CNPq, Hospital de Câncer de Barretos, Barretos, SP, Brazil; d Department of Clinical Oncology, Breast and Gynecology Division, Hospital de Câncer de Barretos, Barretos, SP, Brazil

**Keywords:** Head and neck neoplasms, Quality of life, Translating, Oral health, Neoplasias de cabeça e pescoço, Qualidade de vida, Tradução, Saúde bucal

## Abstract

**Introduction:**

Patients submitted to radiotherapy for the treatment of head and neck cancer have several symptoms, predominantly oral. The Vanderbilt Head and Neck Symptom Survey version 2.0 is an American tool developed to evaluate oral symptoms in head and neck cancer patients submitted to radiotherapy.

**Objective:**

The aim of the present study was to translate the Vanderbilt Head and Neck Symptom Survey version 2.0 into Brazilian Portuguese and cross-culturally adapt this tool for subsequent validation and application in Brazil.

**Methods:**

A method used for the translation and cultural adaptation of tools, which included independent translations, synthesis of the translations, back-translations, expert committee, and pre-test, was used. The pre-test was performed with 37 head and neck cancer patients, who were divided into four groups, to assess the relevance and understanding of the assessed items. Data were submitted to descriptive statistical analysis.

**Results:**

The overall mean of the content validity index was 0.79 for semantic and idiomatic equivalence, and it was higher than 0.8 for cultural and conceptual equivalence. The cognitive interview showed that patients were able to paraphrase the items, and considered them relevant and easily understood.

**Conclusion:**

The tool was translated and cross-culturally adapted to be used in Brazil. The authors believe this translation is suited for validation.

## Resumo

**Introdução:**

Pacientes submetidos à radioterapia para tratamento de câncer de cabeça e pescoço apresentam diversos sintomas, com predominância de sintomas orais. O Vanderbilt Head and Neck Symptom Survey version 2.0 é um instrumento americano que foi desenvolvido para avaliar sintomas orais em pacientes com câncer de cabeça e pescoço submetidos à radioterapia.

**Objetivo:**

Traduzir o Vanderbilt Head and Neck Symptom Survey version 2.0 e adaptá-lo culturalmente para subsequente validação e aplicação no Brasil.

**Método:**

Um método de tradução e adaptação cultural de instrumentos foi utilizado, o qual inclui traduções independentes, síntese das traduções, retrotraduções, comitê de especialistas e pré-teste. O pré-teste foi realizado em 37 pacientes com câncer de cabeça e pescoço divididos em quatro grupos para avaliar a relevância e entendimento dos itens. Dados foram submetidos à análise estatística descritiva.

**Resultados:**

A média geral do índice de validade de conteúdo foi 0,79 para as equivalências semânticas e idiomáticas; e maior que 0,8 para as equivalências cultural e conceitual. A entrevista cognitiva mostrou que os pacientes foram capazes de parafrasear os itens e os consideravam relevante e de fácil entendimento.

**Conclusão:**

O instrumento foi traduzido e adaptado culturalmente ao Brasil. Nós acreditamos que esta tradução está apta para a validação.

## Introduction

Head and neck cancer (HNC) includes tumors that affect the lips, oral cavity, oropharynx, nasopharynx, hypopharynx, larynx, nasal cavity and paranasal sinuses, thyroid gland, and salivary glands.^1^ These cancers account for approximately 10% of malignant tumors.^2^ In Brazil, for the years 2012/2013, approximately 20,000 cases were estimated only for tumors in the oral cavity and larynx.^3^

Therapeutic options for HNC, including radiotherapy, contribute to significant adverse symptoms and loss of function.^4^ Such symptoms may occur immediately, soon after the treatment, or may appear later.^5^ The oral alterations are prominent, and include mucositis, dysphagia, taste and mucosal sensitivity alterations, xerostomia, teeth alterations, and excess mucus.^5^, ^6^, ^7^, ^8^, ^9^ With the exception of xerostomia, mucositis, and dysphagia, these alterations have been little discussed in the literature and are believed to be underreported.^9^ Half of surviving HNC patients have problems and complications five years after the primary treatment, which include pain, problems with teeth, problems with chewing and swallowing,^10^ or high scores for symptoms such as xerostomia, mucus production, and swallowing alterations related to treatment.^11^

The xerostomia resulting from HNC treatment causes damage to oral health^12^ and has a negative impact on the quality of life (QoL) of these patients.^6^ In addition to hyposalivation, which contributes to the onset of mucositis, some patients may have an excessive amount of mucus^11^ that obstructs the airway, resulting in alterations in sleeping, coughing, and choking.^9^ Another common symptom in patients treated for HNC is the alteration of taste,^8^ a direct result of the effect of radiation on the taste buds, and alterations in saliva.^13^ HNC treatment also contributes to a worsening of dental health.^14^ Patients submitted to radiotherapy had worse dental status when compared to those submitted to chemotherapy.^15^ In the Brazilian population, a longitudinal study showed that in patients with oral cancer, the most common problems were related to difficulties in chewing, swallowing, pain, and reduced salivary flow, suggesting the importance of dental monitoring of patients in all stages of treatment.^16^

Some of the toxicities associated with treatment for patients with HNC can be minimized, but are inevitable, highlighting the importance of identifying and controlling adverse effects related to treatment by the health team.^8^ The tools to identify and evaluate these alterations resulting from the treatment can serve as diagnostic tools, helping to establish the most appropriate conduct for the care plan of these patients.

Symptom assessment is often considered within the physical and functional domains of QoL evaluation questionnaires, so there is some difficulty in differentiating symptom research tools from QoL assessment.^17^ In patients with HNC, the most used and validated tools in Brazil to assess QOL are the University of Washington QOL (UW-QOL),^18^ the FACIT-HN,^19^ and the EORTC-HN35,^20^ whereas for symptom research in HNC it is the MDASI-HN.^21^ Overall, the tools used for QOL assessment comprise problems such as dysphagia, xerostomia, and mucositis. However, they fail to report oral symptoms such as mucosal sensitivity, excess mucus, dental problems, and their functional implications. The Vanderbilt Head and Neck Symptom Survey (VHNSS) 2.0^9^, ^22^, ^23^ is an American symptom assessment tool specifically used in patients with HNC whose treatment includes radiotherapy that more broadly assesses oral health components, with a specific domain for dental health and its functional implications. It contains 50 items distributed into 13 domains: nutrition, swallowing/food intake, xerostomia, mucositis, pain, excess mucus, speech/communication, hearing, taste and smell alterations, dental health, mucosal sensitivity, and range of motion. The answer options are graded on a scale of 0–10, so that zero (0) signifies no problem and ten (10) is the maximum presence of a specific problem. The reliability measured by Cronbach's *α* is suitable, ranging from 0.70 to 0.94.^22^ Therefore, the VHNSS 2.0 was selected for translation and cross-cultural adaptation into Brazilian Portuguese.

## Methods

**Figure 1:**
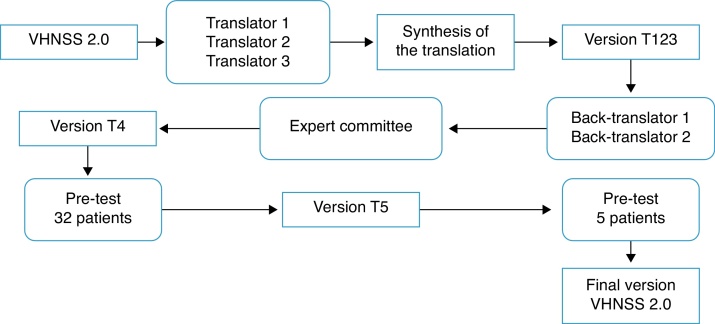
Methodological steps used in the translation and cross-cultural adaptation of the Vanderbilt Head and Neck Symptom Survey (VHNSS) version 2.0.

This was a descriptive, cross-sectional study, using a method of translation and cross-cultural adaptation of the assessment tool, performed in the following five steps:

*Translation and cross-cultural adaptation process*: The process of translation and cross-cultural adaptation of the VHNSS 2.0 into Brazilian Portuguese was performed according to the international guidelines.^24^, ^25^ Consent and authorization in writing was obtained via email from the author of the original tool, Dr. Barbara A. Murphy, and the study was approved by the Research Ethics Committee (protocol No. 644/2012). Fig. 1 summarizes the steps of the translation and cross-cultural adaptation process.

*Translation*: Carried out by three native Brazilian individuals fluent in English, of whom two were from the healthcare area (physicians), whereas the third translator was not.

*Synthesis of translations*: The three translated versions were synthesized into a single version (T123), in a consensus meeting attended by three of the authors (EMB, CEP, and BSRP).

*Back translation*: Performed by American Journal Experts, a company specialized in translations, which received and complied with the guidelines that the two back-translators should be native American English speakers and should be unaware of the original version. The back translations were evaluated and forwarded to the author of the original tool.

*Expert Committee*: The versions were evaluated by an expert committee in the field of health assessment tools, consisting of a head-and-neck oncology surgeon, two clinical oncologists, one nurse, one dentist, and a university professor of linguistics. The aim of the committee was to assess the translated version and compare it to the original one, regarding semantic, idiomatic, cultural, and conceptual equivalence, scoring “1” (one) for the equivalent items, “0” (zero) for the items they did not know, and “−1” (minus one) for the non-equivalent items. They also had autonomy to suggest cultural changes they deemed important. The equivalence calculation was made using the mean of the items of the content validity index (CVI) for each one of them, considered equivalent when >0.8.^26^ Additionally, the suggestions made by the committee were taken into consideration, discussed at a meeting attended by four of the authors (EMB, CEP, BSRP, JSN), which yielded a new version (T4).

### Pre-test

In order to assess the clarity and understanding of the T4 version, a pilot test was performed, which included patients with HNC (oral cavity, larynx, oropharynx, and/or hypopharynx) had been submitted to oncologic treatment, including radiotherapy, for more than six months previously and who agreed to participate by signing the informed consent. Patients with cognitive or mental impairment that prevented them from providing correct information were excluded. Sociodemographic information was obtained through an interview and clinical data from medical records. The 32 patients who met the inclusion criteria were divided into four groups based on domains,^27^ with the first group evaluating items 1–17, the second group items 18–31, the third group items 32–43, and the fourth group items 44–50. Patients answered a structured interview individually to assess the importance and understanding of items and answers, whether they would ask the question differently, and the meaning of the item,^27^ thus ensuring that each item was adequate and understood by the patients. Furthermore, five patients were enrolled at this stage to test the cultural adaptations of six items (5, 10, 14, 27, 38, 43), as the question related to the meaning showed they were not clear for these patients.

### Statistical analysis

Data are shown through descriptive statistics for sociodemographic and clinical characteristics, as well as for the CVI, using SPSS v. 20 software.

## Results

The process of translation and cross-cultural adaptation of the VHNSS 2.0 tool into the Brazilian Portuguese language is described below. It is noteworthy that the back-translation process showed that the initial version in Portuguese corresponded to the English version. The suggestions given by the expert committee were discussed and incorporated into the tool in order to adapt it to Brazilian culture.

Table 1, Table 2 describe the main cultural adjustments within the items and tool responses. The main alteration was the person: in the original tool, all items are in the 1st person singular, whereas the translated version was adapted to the 2nd person singular to provide more clarity and allow the tool to be both self-applied, as well as to be applied by an interviewer.

**Table 1:** Modifications suggested for VHNSS 2.0 items by the expert committee and translators.

Item	Original version	Adapted version	Justification
Title	*Survey* (pesquisa)	*Questionnaire* (questionário)	More adequate word for the Brazilian culture
1st Instruction	*Appropriate Box* (caixa apropriada)	*Chosen option* (opção escolhida)	Clearer and more informal
1	*I currently have a feeding tube in place* (Eu atualmente tenho um tubo de alimentação colocado)	*Are you using a tube to be fed?* (Você está usando uma sonda para se alimentar?)	Clearer and more informal
2nd Instruction	*…In general, a “0” indicates the least amount of problems with a particular symptom and “10” indicates the most problems*. (Em geral “0” indica a menor quantidade de problema com sintoma particular e “10” indica o maior dos problemas)	*In general “0” indicates the absence of a symptom (problem) and “10” indicates the maximum presence of symptom (problem)* (Em geral, “0” indica a ausência de um sintoma (problema) e “10” indica a presença máxima de um sintoma (problema)	The substitution of the term “amount” by “presence” makes the sentence most objective
3	“*like Ensure™ or Boost™*” *(como Ensure™ or Boost™*”*) e* *Liquid Supplement* (suplemento líquido)	“*like Ensure™, Nutren™, Sustagem™” (como Ensure™, Nutren™, Sustagem™)* *Dietary liquid supplement* (suplemento alimentar liquido)	Products available in Brazil. Dietary liquid supplement was added, as it is known in Brazil
5	*Trouble* (problema)	*Difficulty* (dificuldade)	Adaptation to item 4
6	*Trouble* (problema)	*Difficulty* (dificuldade). Removal of the word thin (ralos) and Ensure, substituted by juice (sucos)	Removal of the word thin, which is not commonly used in Brazil and substitution of Ensure by juice, a typical Brazilian drink
15	*Swallowing* (deglutição)	*Swallow* (engolir)	More informal translation of deglutition
16	*Ability to sleep* (capacidade de dormir)	*Sleep* (sono)	Makes the item more objective
17	*Ability to talk* (capacidade de falar)	*Talk* (falar)	Makes the item more objective
18, 19, 20, 21	*Mucous* (muco)	*Secretion* (secreção)	More informal word
27	*Average* (média)	Remoção da palavra *average* (média)	Makes the item more objective
32	*Hearing* (audição)	*Hear* (ouvir)	More informal word, culturally appropriate
34	*I have less desire to eat* (Eu tenho menos desejo de comer)	*You feel less like eating* (Você sente menos vontade de comer)	More informal word, culturally appropriate
37, 38	_	An explanatory parenthesis on ability to smell (capacidade de sentir cheiro) was added	Facilitates understanding
39, 40, 41, 42	*Non applicable* (não aplicável)	*Cannot be applied* (não se aplica).	Facilitates understanding
44, 45, 46, 47, 48	_	Remoção da palavra *lining* (mucosa)	Technical word of difficult understanding, culturally appropriate
48	_	The expression “Cannot be applied” (não se aplica) was added.	Because eventually someone might not have any teeth and the item does not apply
49	*Limitations in the ability* (limitação na capacidade) and jaw (mandíbula)	*Difficuly* (dificuldade) and *mouth* (boca)	Word of difficult understanding, culturally appropriate
50	*Limitations in the ability* (limitação na capacidade)	*Difficuly* (dificuldade)	Culturally adequate

VHNSS 2.0, Vanderbilt Head and Neck Cancer Symptom Survey version 2.0.

**Table 2:** Modifications suggested for the answers to VHNSS 2.0 by the committee of experts and translators.

Answers to items	Original version	Adapted version	Justification
22	*No pain/severe pain* (Nehuma dor/Dor intensa)	*Never/Always* (Nunca/Sempre)	The item refers to the fact of having or not lesions and not the intensity of pain. The answer was adequate to the question
32, 39	*None/Severe* (Nenhum/Grave)	*None/A lot* (Nenhum/Muito)	The word “grave” would not be appropriate in this context, culturally adequate
42	*Not at all/Severe* (Nenhum/Grave)	*None/A lot* (Nenhum/Muito)	The word “grave” would not be appropriate in this context, culturally adequate
14,49,50	*Never/Severe* (Nunca/Grave)	*Never/Always* (Nunca/Sempre)	Answers mixed the concepts of frequency and intensity. It was decided to maintain only the frequency concept

VHNSS 2.0, Vanderbilt Head and Neck Cancer Symptom Survey version 2.0.

The CVI, which was calculated as the mean of the items for each equivalence given by the raters, was 0.79 for idiomatic and semantic equivalence of the items and >0.8 for the other equivalences (Table 3).

**Table 3:** Mean of the CVI of the items.

Equivalence	CVI of items	CVI of answers to items
Semantic/idiomatic	0.79	0.96
Cultural	0.86	0.98
Conceptual	0.89	0.98

CVI, content validity index.

### Pre-test interview

A total of 37 patients participated in this stage, with a median age of 60 years, of whom 32 (86.5%) were males, 21 (56.8%) Caucasians, 27 (73%) married, 26 (70.3%) were barely literate or had not finished elementary school, 23 (62.2%) were from the state of São Paulo, 31 (84%) were professionally inactive, eight (21.6%) were current smokers and 27 (73%) ex-smokers, two (5.4%) consumed alcohol and 21 were ex-alcohol drinkers (56.8%), 29 (78.5%) were Catholics, and 24 (65%) had no associated comorbidity. The clinical characteristics are described in Table 4.

**Table 4:** Clinical characteristics of patients participating in the pre-test.

Variable	Frequency (*n*)	Percentage (%)
*Histological type*		
SCC	37	100.0

*TNM*		
I	3	8.1
II	2	5.4
III	15	40.5
IV	16	43.2
“Missing”	1	2.7

*Location*		
Oral cavity	6	16.2
Hypopharynx	4	10.8
Oropharynx	13	35.1
Larynx	13	35.1
Oral cavity and larynx	1	2.7

*Surgery*		
Yes	21	56.8
No	16	43.2

*Lymphadenectomy*		
No	6	31.6
Yes	13	68.4

*Chemotherapy*		
No	11	29.7
Yes	26	70.3

*ECOG*		
0	26	70.3
1	11	29.7

TNM, classification of malignant tumors; ECOG, Eastern Cooperative Oncology Group.

At the first moment, 32 patients participated in the pre-test and were divided by domain and age, answering questions related to the understanding of the items, with each group containing eight patients, equally divided between those aged up to 60 years old or older. The patients considered the items important, easily understood, and were able to understand the answers. In six items (5, 10, 14, 27, 38, 43) with the question “Could you tell me in your own words what it means for you?”, 25% of patients understood incorrectly. Therefore, these were discussed at a consensus meeting and based on the comments, they were reformulated as follows:

Items 5 and 10: removal of the word “solid”, as patients did not understand what solid was.
Item 14: substituting the word “problem” with “feeling,” as they considered the dry mouth a feeling and not a problem.
Item 38: change of the order of words in the sentence to make it more direct.
Items 27 (“The relief of your pain with analgesics had been: Not applicable, since I do not use analgesics”) and 43 (“Have you had problems with your dentures? Not applicable, because I do not wear dentures”) were not changed, because the authors that participated in the consensus meeting (EMB, CEP, BSRP) considered that the suggestions made by patients to these two items did not add relevant information. These items were reapplied to five patients to confirm that the changes were appropriate. Therefore, the process of translation and cross-cultural adaptation ended, resulting in the Brazilian Portuguese version of the VHNSS 2.0 tool, which in Brazil is known as the “Questionário de sintomas em Câncer de Cabeça e Pescoço de Vanderbilt” (VHNSS 2.0).

## Discussion

The method used in this study allowed the translation and cross-cultural adaptation of the VHNSS 2.0 tool to the Brazilian culture and will allow its use in the assessment of oral symptoms related to treatment that includes radiotherapy of patients with HNC and their functional implications. It is worth mentioning that this is the first tool in Brazilian Portuguese that includes a domain that evaluates the dental status in this population.

During the process of cross-cultural adaptation of the tool, the change from the first to the second person singular resulted in a tool that can be self-applied or applied by an interviewer, as a previous validation study in Brazil showed that 77% of Brazilians prefer assessment tools to be applied by interviewers, with the given reasons being mainly personal preference and difficulty reading.^28^ Additionally, the influence of social and educational levels on HNC incidence must be considered. A meta-analysis study of 41 articles evaluated the association between oral cancer and socioeconomic status, and found that individuals of low socioeconomic status, including low educational level, lower income, and lower occupational class, are more likely to have the disease.^29^

In Brazil, the incidence of HNC is 2.5 higher in individuals who have low educational level.^30^ A study carried out in São Paulo found that 45.4% and 43.6% of patients with HNC were illiterate or had not finished elementary school in the years 2000 and 2006, respectively.^31^ Therefore, a tool that can also be applied by an interviewer meets this population's needs, which in this study comprised the 70.3% of patients who were barely literate or had not finished elementary school, with a median income of one minimum wage.

The reason that led to the translation and cross-cultural adaptation of a new tool was primarily the fact that the available tools in Brazil that evaluate symptoms related to treatment in patients with HNC are associated with tools that assess health-related QoL, and do not include a complete assessment of dental and oral health and their functional implications. It is noteworthy that a detailed oral health assessment is important, since patients treated for HNC, including radiation therapy, often have oral alterations. Additionally, it must be considered that developing new tools takes time and costs money.^32^

Symptoms such as dysphagia and xerostomia have a negative impact on health-related QoL.^6^ Xerostomia is a frequent and important symptom, reported by 52% of patients treated for oral and oropharyngeal cancer; moreover, the functional outcome measured by the Mandibular Function Impairment Questionnaire (MFIQ) is influenced by the incapacity to wear dental prostheses.^33^ It is noteworthy that 40.7% of individuals reported chewing difficulties attributed to their teeth or dentures, 50% reported that their teeth are sensitive to heat, cold, or sweets, and 36% said they had frail or chipped teeth.^9^

Murphy et al. reported that 76% of patients undergoing chemoradiotherapy or radiotherapy for HNC had severe pain in the mouth and throat, resulting in loss of function and increased use of opioids for pain reduction associated with mucosites.^34^ Dental problems affect a large number of patients and occur due to the reduction in the salivary flow directly associated with alterations in dental structures (enamel, dentin) caused by radiation.^35^

The translation and cross-cultural adaptation method used in this study has been consolidated in literature.^24^, ^25^ The translation process involved individuals from the health area, as well as a lay person, so that the terms could be translated to facilitate the understanding by the patients who will answer the tool. The choice of using the services of a specialized company for the back-translation was made to optimize the process with professionals who are native English speakers and are also fluent in Portuguese. The assessment of equivalence by the expert committee showed that the Portuguese version of the VHNSS 2.0 is equivalent to the English version.

One limitation of this study is the small sample size used in the pre-test and the division by domains of the applied structured interviews. The low educational level of the patients with HNC in Brazil may hinder the understanding of the questionnaire, limiting its self-application, although the tool also has been adapted to allow its application by an interviewer.

## Conclusion

Translation and cultural adaptation of the VHNSS 2.0 tool into Brazilian Portuguese has been performed, providing an important tool to assess oral symptoms in patients with HNC submitted to treatment that includes radiotherapy. The results demonstrated that the Brazilian version of the VHNSS 2.0 tool is equivalent to the original in English, was easily understood by patients, and was also adapted to Brazilian culture. Therefore, the tool is considered adequate for the validation step, a process that is underway.

## Authors’ contributions

EMB contributed to the study design, data collection, and writing of the manuscript; ALC contributed to the study design, analysis of equivalence, and reviewing of the manuscript; CEP contributed to the study design, participated in the discussions in the consensus meetings, data analysis, and study review; JSN participated in the committee and discussions at the consensus meetings and contributed with manuscript review; and BSRP contributed to the study design, participated in the discussions in the consensus meetings, and contributed to data analysis and study review. All authors read and approved the final manuscript version.

## Funding

This study was supported by Fundação de Amparo à Pesquisa do Estado de São Paulo (10.13039/501100001807FAPESP, Brazil) Case No. 2012/16768-2.

## Conflicts of interest

The authors declare no conflicts of interest.
